# Investing in success: student experiences in a structured, decelerated preclinical medical school curriculum

**DOI:** 10.3402/meo.v20.29297

**Published:** 2015-09-15

**Authors:** Cindy G. Arvidson, Wrenetta D. Green, Renoulte Allen, Christopher Reznich, Brian Mavis, Janet R. Osuch, Wanda Lipscomb, John O'Donnell, Patricia Brewer

**Affiliations:** 1 Office of Preclinical Curriculum, College of Human Medicine, Michigan State University, East Lansing, MI, USA; 2 Office of Preclinical Curriculum, College of Human Medicine, Michigan State University, Grand Rapids, MI, USA; 3 Office of Medical Education Research and Development, College of Human Medicine, Michigan State University, East Lansing, MI, USA; 4 Office of Student Affairs and Services, College of Human Medicine, Michigan State University, East Lansing, MI, USA

**Keywords:** extended curriculum, non-traditional medical students, accommodating life events, academic challenges, career satisfaction

## Abstract

**Purpose:**

Many students in the Michigan State University College of Human Medicine (CHM) are non-traditional with unique needs and experiences. To meet these needs, in 1988 CHM developed a structured Extended Curriculum Program (ECP), which allows students to take longer than 2 years to complete the preclinical curriculum. This work examined the reasons why students extended their programs, their perceptions of that experience, and the outcome with respect to satisfaction and success in their careers after graduation.

**Methods:**

The analysis used data from the college database, follow-up surveys of residency directors and graduates, surveys of graduates who extended, and the AMA Physician Masterfile.

**Results:**

Graduates who responded to the survey were evenly split between those who extended for academic reasons and those who extended for other reasons. Although feelings about extending were mixed at the time of extension, nearly all respondents agreed that extending was the right decision in the long run. Extended students continued to face academic challenges having lower basic science averages, lower USMLE Step 1 and 2 first attempt pass rates, and more instances of repeated clerkships compared to those who did not extend, however, most were able to secure a residency in the specialty they desired and had comparable career satisfaction ratings.

**Conclusions:**

The ECP allows some students to complete medical school who otherwise may not have been able to do so. This analysis has provided valuable information that was used to improve the program, allowing CHM to continue its mission of training a diverse set of students to be exemplary physicians.

The core of the mission of the Michigan State University (MSU) College of Human Medicine (CHM) is the goal of educating physicians to provide service at home and abroad, promoting the dignity and inclusion of all people, and responding to the needs of the medically underserved (1). CHM was ranked sixth among US medical schools with respect to its ‘social mission’, based on a composite score including percentage of graduates who practice primary care, who work in health professional shortage areas, and are underrepresented minorities (2). The founding vision for CHM of a medical school for Michigan students to meet the healthcare needs of Michigan residents was grounded in MSU's land-grant philosophy (3). In order to achieve the state legislature's mandate to diversify the workforce, CHM has developed strategies to recruit and retain students with a broad range of interests and experiences (4, 5).

Admission to CHM is determined by evaluating both academic and non-academic metrics including how well the applicants’ backgrounds and experiences align with the mission. Thus, each year we matriculate a diverse group of students, some of whom may be considered non-traditional, economically and/or educationally disadvantaged. Some students will have been non-science majors as undergraduates, some will have been in the workforce for a few years before matriculation, and others will have pursued other life-enriching activities before returning to school. To support its mission, CHM invests a substantial amount of resources in academic and other support for every student.

The current curriculum at CHM can be divided into two parts: preclinical and clinical. The preclinical curriculum is the first 2 years, simultaneously delivered at two preclinical campuses (East Lansing and Grand Rapids) and culminating with the student taking the United States Medical Licensing Examination (USMLE) Step 1 exam (Fig. 1), which must be passed before the student is permitted to continue their clinical education. Clinical training then takes place at multiple community campuses around Michigan. There is flexibility in the preclinical curriculum such that there are a variety of ways a student can extend this schedule beyond 2 years. Since the founding of the college in 1965, CHM has had a tradition of flexibility with respect to length of time allowed from matriculation to graduation, with nearly 25% of our graduates taking more than 4 years to complete the MD degree. (Note: This does not include graduates who were in dual-degree programs.) A well-structured Extended Curriculum Program (ECP) has been in place since 1988.

**Fig. 1:**
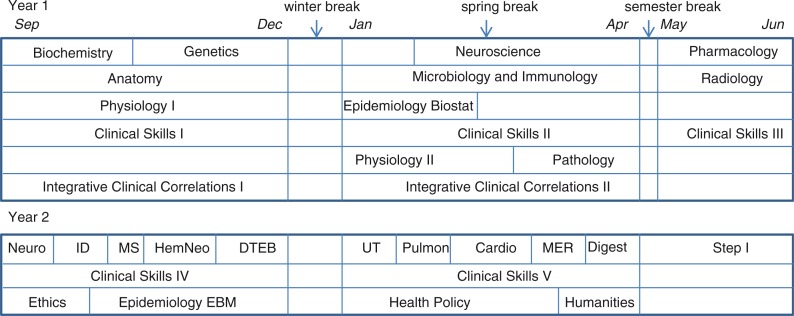
Regular preclinical curriculum. Year 1 consists of two semesters (fall and spring) of 16 weeks followed by a 7-week summer semester. Some courses are full semester while others are shorter. Biochemistry, genetics, epidemiology/biostatistics, and pathology are each 8 weeks long; physiology II is 9 weeks, and neuroscience is 12 weeks. Year 2 consists of two semesters (fall and spring) of 16 weeks. Year 2 courses vary in length, with the problem-based learning (PBL) courses running sequentially to each other, and parallel to clinical skills and courses in ethics (8 weeks), epidemiology/evidence-based medicine (8 weeks), health policy (12 weeks), and humanities (4 weeks). PBL domains: neuroscience (neuro, 3 weeks); infectious disease/immunology (ID, 3 weeks); musculo-skeletal (MS, 2 weeks); hematology/neoplasia (HemNeo, 4 weeks); disorders of thought, emotion, and behavior (DTEB, 4 weeks); urinary tract (UT, 3 weeks); pulmonary (3 weeks); cardiology (4 weeks); metabolic, endocrine, reproductive (MER, 3 weeks); and digestive (Dig, 3 weeks).

Nearly a third of US medical schools offer formal options for decelerating or extending a student's curriculum according to a 2004 report (6). Some of the programs are only for students with academic challenges, whereas others are an option for any student, regardless of academic performance. Interestingly, very few medical schools publicize decelerated options, although references to such options can be found in student doctor and pre-medical discussion forums on the internet. Schools who do publicize decelerated curricular options include George Washington University, Boston University, and University of Minnesota Schools of Medicine. The George Washington University School of Medicine describes a decelerated program that spreads out the first two semesters over 2 years (7). Students in the decelerated program are held to strict academic standards in order to remain in medical school. Boston University School of Medicine describes three variations of their curriculum that allow for more than 4 years to complete the MD degree (8). Two of them are designed for students who wish to pursue activities outside of and in addition to the traditional medical school curriculum and the third is similar in structure, but specifically designed for students needing additional time as a result of academic difficulties. The University of Minnesota publicizes a program called Flexible MD (9). The goal of their program is to allow students to pursue additional activities, outside of the regular medical school curriculum, while in medical school. Some of the additional activities lead to a second degree or certificate, but this is not a requirement. Students must apply to the program, and stringent academic and other criteria are required. Thus, in contrast to many other decelerated programs, this program is not for academically challenged students.

The ECP at CHM allows a student to extend the preclinical curriculum to 3 years or more, as needed. Because CHM policy states that a student must complete requirements for the MD degree within 8 years of matriculation, extension of the preclinical curriculum is limited to no more than 6 years. A student may extend for academic or other reasons, and the procedure and structure is the same for all students in the ECP. The purpose of this paper is to describe the ECP program and document the outcomes of the program from both academic and non-academic standpoints to evaluate its success and needs for improvement as the medical education landscape changes with respect to curriculum design and delivery.

## ECP description

### Identification of students for participation in the ECP

There are three ‘pathways’ that may lead to a student extending their preclinical curriculum. Each required course in the preclinical curriculum is offered once per year and all students take all classes together. Thus, the first pathway is required extension for academic reasons to repeat one or more courses. A student who does not pass a required course and subsequently does not pass a remediation opportunity will have to extend their curriculum in order to repeat the course at its next offering. These extensions typically occur early in the semester immediately following the course(s) that was/were not passed. Another circumstance that can lead to a required academic extension is when a student struggles in multiple courses. CHM policy states that any student that accumulates three unremediated, non-passing grades at any time is required to extend their curriculum, regardless of whether the student ultimately remediates the courses. The goal is to redistribute the academic burden to promote academic success and is not meant to be punitive, but rather supportive of the student.

The second pathway is voluntary extension for academic reasons. There are students who are passing their required courses but feel they could perform better academically with more time. CHM uses a pass/no-pass grading system to discourage competition and encourage students to work together. However, because residency placement depends in part on scores on USMLE Step 1, and the preclinical curriculum is preparation for Step 1, students strive to do well in the preclinical courses to assure themselves that they will be ready to excel on USMLE Step 1. Students may voluntarily extend at any time during the preclinical curriculum and most students choose to extend early in a given semester when dropping a course will not result in a grade being reported.

The third pathway is for those students who wish to extend for purely personal reasons. Examples include students who experience health problems (personal or family member), students who started a family before entering medical school and want to maintain a balance to spend more time with their children during their early formative years, students who were in the workforce between undergraduate and medical school who want to continue some level of participation in those activities, and students who wish to have extra time to pursue other life-enhancing activities. We have had students extend to tour as a member of a rock band, train for the Olympics, and simply to take time to enjoy life and travel, which they did not feel they had time for when preparing for medical school.

### Engaging students in the ECP

A student may choose to extend anytime during the preclinical curriculum and the process is the same regardless of the time or the reason. The curriculum designed for each extended student is unique and is developed in consultation with selected administrators in the Office of Preclinical Curriculum (academic team meeting). The student's individual needs and background are taken into account when developing the plan. These are important considerations that align with our philosophy of encouraging cohesiveness among our students and preventing feelings of isolation.

**Fig. 2:**
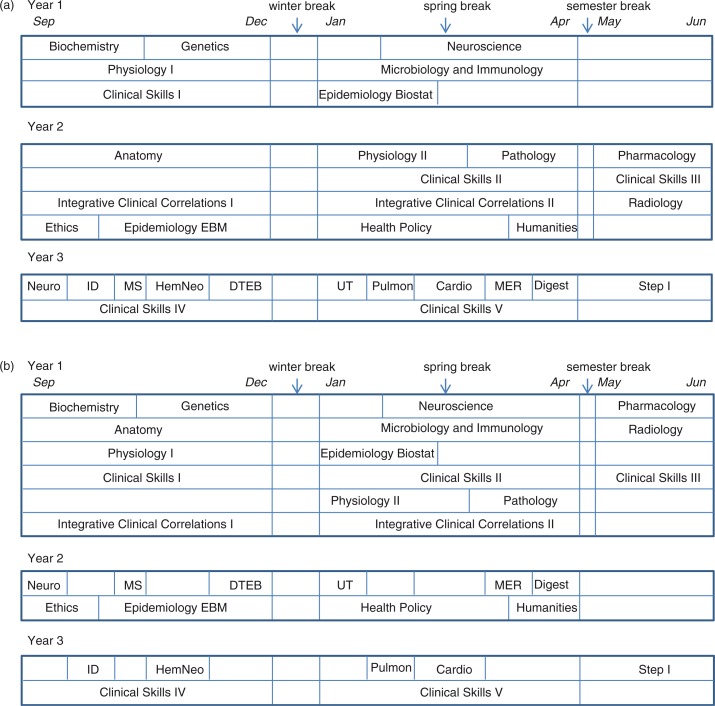
(a) Sample year 1 extended curriculum. A student extending in early in the first year can spread out the year 1 courses over 2 years, with the following restrictions: either physiology I or anatomy must be taken in the first year; physiology I is a prerequisite for physiology II; biochemistry is a prerequisite for microbiology/immunology; microbiology/immunology and physiology II must be taken prior to or concurrently with pathology; and pharmacology must be taken the summer immediately prior to the first PBL course (neurology). In the second year, a student may take one or more of four-year 2 courses (ethics, epidemiology/EBM, health policy, and humanities). (b) Sample year 2 extended curriculum. A student extending in year 2 (after completing year 1) can spread out the year 2 courses over 2 years, with the following restrictions: the first PBL domain, neuroscience, must be taken before all other; pulmonary and cardiology should be taken in the same year; and clinical skills IV and V must be taken in the year immediately prior to Step I.

For a student who extends early in the preclinical curriculum (during year 1), the plan would spread out the 13 courses normally taken in year 1 over 2 years. There is some flexibility in the sequencing of these courses, although some sequencing needs to be maintained for optimal progress through the curriculum. A student who elects to take clinical skills II or III in year 1 must audit those courses in year 2 to keep the skills fresh for clinical skills IV and V. In addition to the year 1 courses, there are four courses normally taken in year 2 of medical school (medical ethics, epidemiology/evidence-based medicine, health policy, and medical humanities), which can be taken during year 2 of an extended curriculum. The third year of this extended curriculum includes the problem-based learning (PBL) series as well as the last two semesters of a five-semester clinical skills curriculum. A sample year 1 extended curriculum is depicted in Fig. 2a.

A second option for extending is to complete the regular year 1 curriculum and then spread the year 2 curriculum over 2 years. In this plan, the PBL series is divided such that half of the 10 PBL courses (domains) are taken in year 2 of the student's curriculum and the remainder taken in year 3. As in a year 1 extension, there is some flexibility in which domains are taken in which year. The year 2 clinical skills courses are typically taken in the last (third) year to optimize student readiness for clinical clerkships. A sample year 2 extended curriculum is depicted in Fig. 2b.

### Financial considerations

The tuition structure for medical students at MSU is, in part, based on the number of credits enrolled in a given term. Thus, careful attention is given to financial consequences of an extended curriculum and schedules are adjusted whenever possible to minimize the impact. MSU also established a tuition plan option (ECP tuition) for medical students who extend their curriculum during their preclinical studies. Depending on the timing of the extension and whether or not there are courses that must be repeated, ECP tuition may or may not save money compared to the regular tuition option. Students are counseled by the MSU Office of Financial Aid as part of the extension planning process. The decision to change to ECP tuition is a onetime opportunity and a student cannot go back and forth between regular and ECP tuition during the preclinical curriculum. In general, a student that follows one of the extended options in Fig. 2 will pay 12% more in tuition on the regular tuition plan and 11% more on ECP tuition compared to a regular 2-year curriculum. Of course, this does not take into the account that there will also be an additional year of living expenses, which is sometimes an added burden for an extended student but is unavoidable.

### Resources/academic support

Although many schools offer decelerated/extended curricular programs, more than half of those offer no additional support to those students other than the extra time afforded by extension (6). This is not the philosophy at CHM. A guiding principle of the ECP is maintaining student engagement. Just adding time to complete the preclinical curriculum is not sufficient to allow students to reach their full learning potential and prepare them adequately for the USMLE Step 1 examination and their clinical clerkships.

CHM provides a variety of *Learning Enhancement Academic Programs* (LEAP) (10) to help all students perform at a mastery level, defined as attaining 85% and above on course examinations. These are highly structured, interactive learning programs with activities that are based on the Piagetian premise that learning is not the same as making a mental copy of something: *To learn, one must interact with the material or act upon it. Students explaining the material to each other is a way of manipulating it, a way of knowing it* (11). These programs include one-on-one tutoring for individual courses, group study for individual courses, and groups that allow students to review and preview multiple courses as needed throughout their preclinical years. The latter style was designed specifically for extended students and allows students who have already completed a course to review material while assisting others who are actively taking the course thereby encouraging peer teaching. The goal is to ensure that students are connected with their classmates as well as all available resources.

We use a holistic approach in providing support for our students. We focus both on content acquisition and skill improvement. Although the curriculum determines a student's content learning needs, the academic support team has developed a systematic approach to addressing skill development. During orientation, entering students take a *Learning And Study Strategies Inventory* (LASSI) (12) to determine strengths and weaknesses in their study approaches. Using LASSI results and a variety of academic and non-academic characteristics, we help students develop a plan to improve the skills needed to be successful. Toward that end, we implement programming for learning skill enhancement focusing on specific skills, including critical thinking/problem-solving, test-taking, note-taking, and time management.

Tutors and group leaders include more advanced medical students, graduate students, residents, physicians, and Master's or PhD-level content experts. We also have physicians who help students practice clinical skills they may have begun to forget. Students in the ECP are encouraged to participate in at least one LEAP group and those who extend for academic reasons are required to participate in some form of academic support. Through an individualized, skill-focused program we are able to address all of the learning needs of our ECP students.

## Methods

A plan was developed to evaluate the ECP and student outcomes associated with the program, which was reviewed by the MSU Institutional Review Board. Three data sources were used.

### College database

The student information database maintained by the college registrar's office was queried to determine when and how many students extended their curriculum. The target sample was all matriculants from 1991 to 2003. Using this list of extended students, outcomes related to subsequent academic performance were compared to non-extended students. In addition, comparisons of the early career characteristics of extended and non-extended graduates were based on our institutional follow-up surveys of residency directors for graduates 1 year after graduation (PGY1), and the graduates themselves 2 years post-graduation (PGY2). The academic and career information for extended and non-extended students was obtained from the Student Performance and Outcomes Database, which is maintained by the Office of Medical Education Research and Development to support program evaluation. This database contains information about all CHM matriculants from their admission application through their academic performance in medical school as well as information about residency and career choice.

### Survey of extended graduates

All students who matriculated between 1991 and 2003, extended during the preclinical years, and who ultimately graduated from medical school, were contacted to participate in a survey. Not included in this group were students in structured dual-degree programs (MD/PhD, MD/MS, or MD/MPH) or those that withdrew, transferred, or were dismissed from the college. A paper version of the questionnaire was mailed along with a link for an online version. Non-respondents received up to three reminders each sent 3 weeks apart. The questionnaire focused on four major themes: 1) reasons for extending, 2) emotional reactions to extending, 3) perceived impact of extension on their subsequent medical school education, and 4) perceived impact of extension on residency placement and career satisfaction.

### AMA Physician Masterfile

Career characteristics were obtained from the AMA Physician Masterfile data (13).

## Results

### Extended student characteristics

Of the CHM students who matriculated from 1991 to 2003, 215 (15%) extended their curriculum during the first 2 (preclinical) years of medical school. When compared to non-extended students (*N*=1,189), extended students (*N*=215) tended to be older at matriculation (27.1 vs. 24.6 years; *t*=5.79, *p*<0.001), were more likely to be women (63 vs. 52%; chi-square=7.31, *p*=0.007), and more likely to be members of racial/ethnic groups underrepresented in medicine (37 vs. 15%; chi-square=49.74, *p*<0.001).

Of the 215 matriculants who extended, 182 (85%) graduated, 23 (11%) were subsequently dismissed from medical school, and 10 (5%) withdrew or transferred to another school. In this study, we focused on the 182 graduates who participated in the ECP.

### Graduate survey

Of 182 graduates who participated in the ECP, 79 (43%) responded to the survey.

#### Reasons for extending

When asked why, 38% of the graduates who extended indicated they extended for academic reasons. Some of these were voluntary and others were required, likely to repeat a course they did not pass. Other reasons for extending included personal or family health reasons (19%), and family needs other than health (26%). Sixteen percent of the respondents indicated that they extended for other reasons, some of which were to finish other professional obligations, work to support family, pursue electives, and learn another language.

#### Perceptions

Table 1 summarizes some of the responses to the survey. These data show that many of the respondents felt good about the decision to extend from the start, although a significant number of them indicated they did not. A majority of them agreed that extending allowed them to enhance their study skills, and the extra time allowed them to more thoroughly learn the material. More importantly, nearly all of them indicated that extending allowed them to address the issues that led to the extension in the first place.

**Table 1:** Graduates’ perceptions of the extended curriculum program^a^

		# Agree or strongly agree (out of 79)
1.	At the time, extending my curriculum was easy for me to accept.	50 (70%)
2.	At the time, I was uncertain about whether or not extending my curriculum was a good idea.	31 (43%)
3.	Extending my curriculum gave me time to improve my study skills.	53 (77%)
4.	Extending my curriculum gave me time to learn course material more thoroughly.	56 (81%)
5.	When I was in medical school I felt there was a certain stigma attached to being an extended student.	43 (60%)
6.	After I extended my curriculum I felt less connected with other medical students.	41 (57%)
7.	I felt that extending my curriculum allowed me to address the challenges that led to my extension.	65 (92%)
8.	The impact of curriculum extension on my preparation for year 3 clerkships:	
	Negative	2 (3%)
	Neutral	25 (35%)
	Positive	45 (63%)
9.	The impact of curriculum extension on securing a residency position:	
	Negative	8 (11%)
	Neutral	41 (58%)
	Positive	22 (31%)

a Respondents were provided with a four-point rating scale, where 1=strongly disagree and 4=strongly agree. Data presented are based on respondents indicating agree or strongly agree to each statement.

Many respondents (60%, Table 1) reported negative feelings about extending in that they felt there was a certain stigma attached to being on ECP. However, a significant number of them (40%) indicated they did not feel there was a stigma attached. Many felt disconnected from other students, because most students follow the same curriculum and are in the same classes all the time. Students who extend end up taking some courses with their incoming cohort and others with the class that enters a year later, leaving them feeling disconnected from both cohorts. However, when asked, many graduates felt that they did have the support of their classmates and most also felt supported by both their families and the college administration.

#### Outcomes

Some of the biggest concerns of students when they extend are how extending will affect board scores, performance in the clinical clerkships, and residency placement. Of the 182 graduates who matriculated between 1991 and 2003 and extended their preclinical curriculum, 77% of them passed the USMLE Step 1 exam on the first attempt (Table 2). This is considerably less than the 96% rate for students who did not extend and matriculated in the same time period who passed the USMLE Step 1 exam on the first attempt. Similar results are seen when USMLE Step 2 (clinical knowledge) exam results are compared with 83% of students who extended passing on the first attempt and 97% of those who did not extend passing on the first attempt.

**Table 2:** A comparison of academic performance and career outcomes for extended and non-extended graduates (based on matriculants from 1991 to 2003)

	Not extended (*N*=1,146)	Extended (*N*=182)	Test statistic and probability
Academic performance			
Year 1 basic science mean	85.0	80.4	*t*=9.56, *p*<0.001
Year 2 basic science mean	85.4	82.9	*t*=7.22, *p*<0.001
USMLE Step 1 pass rate (first try)	96%	77%	Chi-square=96.54, *p*<0.001
USMLE Step 2 CK pass rate (first try)	97%	83%	Chi-square=53.24, *p*<0.001
Needed to repeat all or part of at least one clinical clerkship	20%	53%	Chi-square=91.22, *p*<0.001
Residency program characteristics			
Residency in primary care specialty (%)	55%	54%	Chi-square=0.06, *p*=0.804
Residency in Michigan (%)	49%	55%	Chi-square=2.05, *p*=0.152
Residency director ratings^a^			
General medical knowledge (mean)	4.0	3.6	*t*=4.01, *p*<0.001
Clinical problem-solving (mean)	4.1	3.7	*t*=3.38, *p*<0.001
Clinical skills (mean)	4.1	3.8	*t*=2.86, *p*=0.004
Patient management (mean)	4.1	3.8	*t*=3.30, *p*=0.001
Professional attributes (mean)	4.4	4.4	*t*=0.99, *p*=0.32
Attributes as a learner (mean)	4.3	4.1	*t*=2.26, *p*=0.024
Communication skills (mean)	4.3	4.4	*t*=0.50, *p*=0.62
Written records (mean)	4.1	3.9	*t*=2.26, *p*=0.024
Emergency care (mean)	4.1	3.7	*t*=4.03, *p*<0.001
Overall rating (mean)	4.1	3.8	*t*=3.36, *p*<0.001
PGY2 graduates’ ratings^b^			
Career satisfaction (mean)	4.1	4.1	*t*=0.17, *p*=0.862
Satisfaction with CHM education (mean)	4.2	4.3	*t*=0.74, *p*=0.457
Quality of internship preparation (mean)	3.9	4.0	*t*=0.90, *p*=0.370
Life satisfaction (mean)	4.0	4.1	*t*=0.46, *p*=0.649
AMA Physician Masterfile data			
Practice in-state (%)	41	41	Chi-square=0.06, *p*=0.812
Primary care practice (%)	42	42	Chi-square=0.02, *p*=0.885
Primary practice specialty^c^			
Generalist specialties (%)	30	28	
Medical specialties (%)	20	27	
Surgical specialties (%)	29	21	
Support specialties (%)	22	24	

a Ratings based on a five-point rating scale: 1=substantially below average, 2=below average, 3=average, 4=above average, 5=substantially above average.

b Ratings based on a five-point rating scale: 1=very dissatisfied, 2=dissatisfied, 3=mixed feelings, 4=satisfied, 5=very satisfied.

c Generalist specialties are general internal medicine, family medicine, and pediatrics; medical specialties include subspecialties within internal medicine, family medicine, and pediatrics as well as allergy/immunology, dermatology and its subspecialties, psychiatry and neurology and their subspecialties, occupational medicine, public health and preventive medicine and related specialties, and medical genetics specialties. Surgical specialties include general surgery and its subspecialties, colon and rectal surgery, neurological surgery, obstetrics-gynecology and subspecialties, ophthalmology, orthopedic surgery, otolaryngology, plastic surgery, thoracic surgery, and urology. Support specialties include anesthesiology and critical care, emergency medicine, nuclear medicine, pathology and its subspecialties, physical medicine and rehabilitation, and radiology and related subspecialties.

When asked how prepared they felt for their clinical clerkships, more than half of the graduates who extended indicated that extending had a positive effect, whereas very few indicated that extending had a negative effect (Table 1). Looking at the performance of these graduates in these clerkships, 53% of them needed to repeat all or part of at least one clerkship due to failure of a component of the clerkship (Table 2). This is considerably greater than the proportion (20%) of the non-extended graduates who needed to repeat at least one clerkship. When asked about the effect on securing a residency, the majority of the graduates indicated that extending had no effect, with a significant number indicating that extending had a positive effect on residency placement (Table 1). Few said extending had a negative effect. More importantly, though, nearly all of the graduates (95%) that extended were able to secure a residency in the specialty of their choice. Overall, 92% of the graduates who responded indicated that looking back, extending was the right decision for them.

### Subsequent academic performance

Academic performance indicators for extended and non-extended graduates are compared in Table 2. Extended students had lower basic science scores in their preclinical curriculum compared to non-extended students, had lower initial pass rates for USMLE Step 1 and Step 2 clinical knowledge, and were more likely to need to repeat at least one clinical clerkship.

### Career characteristics

Our institutional follow-up of PGY1 graduates is based on ratings from residency program directors (Table 2). Extended graduates received lower ratings than non-extended graduates for eight of the 10 attributes rated by residency program directors. The small but significant differences between the groups ranged from 0.2 to 0.4, which represents a 5–10% reduction for extended graduates relative to other graduates. There was no difference between the two groups based on the location of their residency program (in-state vs. out-of-state) and whether or not they were in a primary care residency. When surveyed in their second year after graduation, extended graduates’ levels of satisfaction with their medical education, career, and life were comparable to those of non-extended graduates.

With regard to practice characteristics, there were no differences between the groups in primary care practice or in-state practice. Extended graduates had specialty interests comparable to those of non-extended students, with similar distributions of generalist, medical, surgical, and support specialties.

## Discussion

Several US medical schools offer formal or informal options for decelerating or extending a student's curriculum. McGrath and McQuail (6) reported that overall, ~90% of students in the decelerated programs of the schools who responded to their survey went on to complete their MD degree. Their conclusion was that the programs were successful in that students who might have otherwise been dismissed were allowed to remain in medical school and reach their goal of becoming physicians. Because a goal at CHM is to educate and graduate a diverse class of students, flexibility, as is provided by the ECP, is important to achieving this goal. The goals of the ECP are to give students the best opportunity to achieve their full learning potential such that they master the preclinical curriculum and achieve optimal performance on the USMLE Step 1 exam, while also allowing them the flexibility to pursue life-enhancing opportunities and accommodate unexpected life events. Extended students often continue to have academic challenges throughout their undergraduate and graduate education but ultimately achieve satisfaction comparable to their non-extended colleagues. Academic extension does not appear to have an adverse impact in terms of our institutional mission: extended and non-extended graduates were equally likely to practice in-state and choose a primary care specialty. In fact, this curricular option results in a more diverse workforce by supporting the aspirations of disadvantaged and non-traditional students.

From our survey of graduates who extended during the preclinical years, we learned that there were a variety of reasons that students extended their curriculum, with nearly an equal split between academic and non-academic reasons stated by the respondents. Some of the students were not happy with the decision and had negative feelings about the program and process. These were most likely students who were required to extend due to academic difficulties as opposed to doing so voluntarily. Because nearly all students who responded agreed that looking back, extending was the best option for them, the students with negative feelings about having to extend still found a way to turn it into a positive by successfully completing their MD. It should be noted, however, that there is likely a selection bias in our study because we only have comments from graduates who responded, who are more likely to have been satisfied with their experience. The most positive outcome of this study was learning that in spite of extending their preclinical curriculum and their feelings about it, nearly all of the students felt they were able to secure a residency in the specialty of their choice.

From our surveys as well as experiences with the ECP over the years, we have learned some valuable lessons that have led to improvements to the program. Two major lessons learned are 1) the need for clear communication of expectation and options to students who may need to extend their curriculum, and 2) the need for a clearly defined process within the ECP, so that programming decisions have a rational basis and are not approached *ad hoc*. The ongoing need for improvements to the ECP has driven a series of constructive changes. Examples of such improvements include:

1. A detailed written description of the ECP including procedures for extending in year 1 and year 2; considerations such as impact on USMLE performance, scholarships, residency selection and financial impact; and sample extended curricular program schedules are provided to all students at matriculation.
2. Timely communication of the ECP option to students who may face challenges to their ability to complete their MD degree requirements in 4 years.
3. Standard agendas for academic team meetings have been developed to review program requirements for those students extending due to academic performance issues.

As the college prepares to implement a full revision of its medical school curriculum, we will continue to develop improvements to the ECP.

## Conclusions

The ECP allows some students to complete medical school who otherwise may not have been able to do so. As the residency match gets more competitive, it will be imperative that struggling students have opportunities such as the ECP to allow them to pass USMLE Step I the first time and do well on the exam. CHM's investment in these students fits well with CHM's mission of training a diverse group of students, some of whom will care for patients from underserved populations. The ECP also allows some students to complete medical school while also participating in life-enriching experiences that ultimately lead to greater life and career satisfaction.

## Previous presentation

Some of the data in this study were presented at the AAMC Central Group on Educational Affairs Meeting, Cincinnati OH, March 2013.
